# Suspected meningeal infiltration in H syndrome: A case report

**DOI:** 10.1016/j.jdcr.2025.10.048

**Published:** 2025-11-07

**Authors:** Zyad Al-Frejat, Adnan Alghazzawi, Lora Shammas, Marie Ahmad, Dianna Al-Frejat

**Affiliations:** a Department of Radiology, Damascus University, Damascus, Syria; b Department of Neurology, Damascus University, Damascus, Syria; c Damascus University, Damascus, Syria; d Department of Dentistry, Syrian Private University, Damascus, Syria

**Keywords:** cerebral edema, dermatology, H syndrome, neurology

## Introduction

H syndrome is an autosomal recessive disorder characterized by histiocytic proliferation, with both cutaneous and systemic manifestations. The first case of this syndrome was described in 2008 by Molho-Pessach et al in 10 Arab patients descended from 6 different ancestors.^1^

Most described cases of H syndrome have been of Arab descent, but it may also be seen worldwide. This syndrome is caused by mutations in the SLC29A3 gene, which encodes the human equilibrative nucleoside transporter 3 (hENT3).

The human equilibrative nucleoside transporter 3 (hENT3) facilitates sodium-independent transport of nucleosides across lysosomal and mitochondrial membranes and contributes to nucleotide synthesis through salvage pathways. As a result, mutations in this gene can affect multiple organ systems, explaining the diverse clinical manifestations of the syndrome.^1^

The diagnosis of H syndrome can be challenging, especially when characteristic features are subtle or mimic other conditions. A detailed medical history and physical examination, with special attention to family background, are essential, while confirmation requires genetic testing for SLC29A3 mutations.^1^

In this report, we present the case of a young girl diagnosed with H syndrome who exhibited unusual neurological findings. By presenting this case, we aim to enhance awareness of this rare disease.

## Case Report

**Fig 1:**
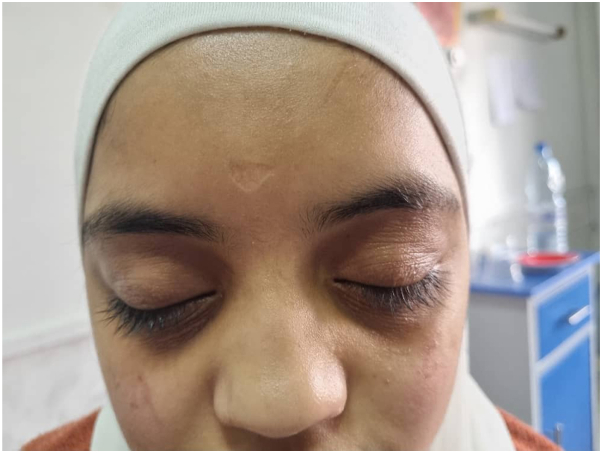
Periorbital edema.

**Fig 2:**
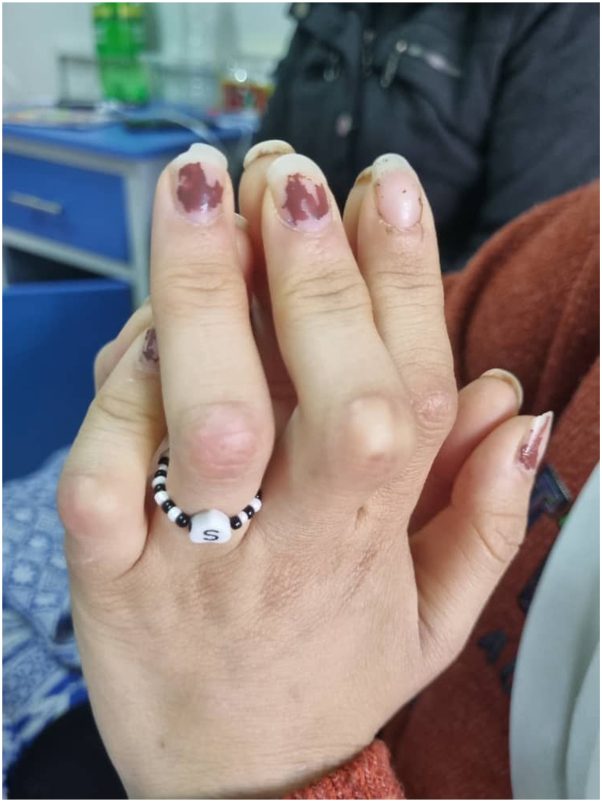
Fixed flexion contractures of proximal second to fifth interphalangeal joints in the patient.

**Fig 3:**
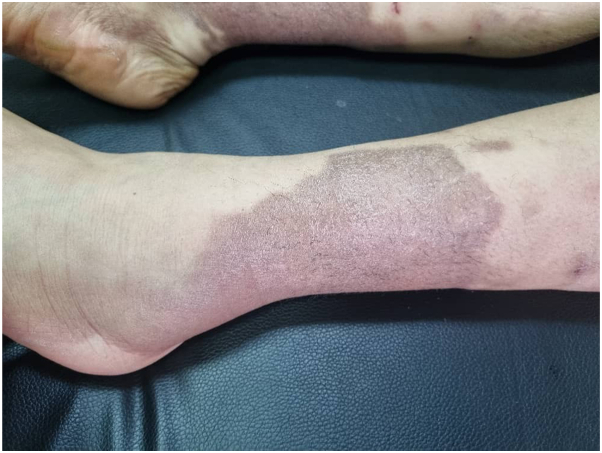
The pigmented sclerosis of the leg.

**Fig 4:**
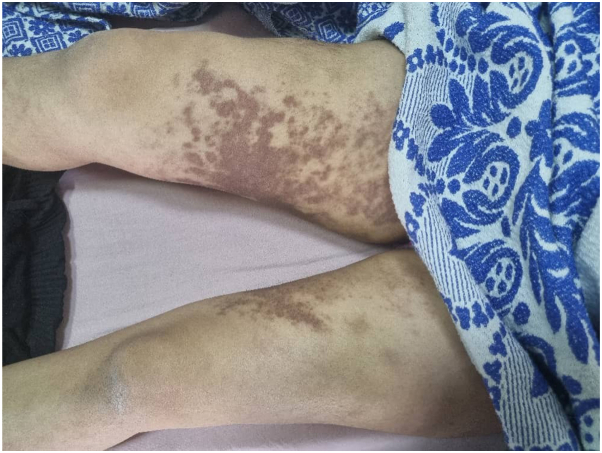
Hyperpigmented-violaceous sclerotic plaques symmetrically involving the bilateral inner thighs, inner legs.

A 16-year-old Middle Eastern female presented to the clinic with a right temporal abscess persisting for 20 days, accompanied by right-sided periorbital edema (Fig 1). Ophthalmic examination revealed bilateral optic disc edema (grade 2), a relative afferent pupillary defect (RAPD) in the left eye, and impaired red color saturation. A brain MRI demonstrated diffuse meningeal enhancement. Physical examination revealed nodules on the proximal interphalangeal (PIP) joints of both hands (Fig 2), diffuse cutaneous lesions on the thighs and legs (Figs 3 and 4), and a chronic moderate frontal headache. Neurologic assessment showed intact cranial nerves, normal motor tone, absent deep tendon reflexes, and bilateral plantar responses graded 2/5.

The patient had a history of poorly controlled type 1 diabetes mellitus (diagnosed in 2010, treated with mixed insulin for the past 2 years, HbA1c = 9.7%), autoimmune hepatitis (treated with prednisolone for 2 years), and hypopituitarism (on hormonal replacement therapy for the past 7 months at 0.3 units/day). Additionally, she had a history of autoimmune demyelinating polyneuropathy and chronic gastritis.

There was no family history of similar cutaneous or systemic conditions, and no known consanguinity between the parents.

Cerebrospinal fluid (CSF) analysis showed clear fluid with elevated glucose (116 mg/dL) and protein (66.7 mg/dL) levels, with no abnormal cells. Complete blood count (CBC) was normal, and autoimmune markers (ANA, ANCA, RF) were negative. Endocrine tests revealed normal thyroid function (T4: 1.4 ng/dL, TSH: 1.35 μIU/mL) and prolactin level (8.1 ng/mL).

Contrast-enhanced CT of the abdomen and pelvis revealed bilateral inguinal lymphadenopathy and a bicornuate uterus, with no other abnormalities.

A biopsy of the cutaneous lesions demonstrated dermal fibrosis with lymphocytic aggregates, histiocytic infiltration, and occasional multinucleated giant cells. These findings confirmed the diagnosis of H syndrome, a rare autosomal recessive disorder characterized by systemic manifestations, including cutaneous, ophthalmologic, and neurologic involvement.

## Discussion

H syndrome is a constellation of symptoms caused by mutations in the SLC29A3 gene, inherited in an autosomal recessive pattern.^2^ The underlying mechanism involves defective histiocyte functions leading to systemic manifestations. The severity and range of symptoms vary depending on the systems affected. It is important to note that the majority of reported cases are of Arab ancestry, with a mean age of diagnosis at 17 years, consistent with our patient’s demographic profile.^3^

Cutaneous plaques are the hallmark of H syndrome, observed in nearly 68% of patients, with 91% of these cases involving the lower limbs, the inner thighs in particular.^3^ While hypertrichosis often accompanies these plaques, it was absent in our patient, highlighting the phenotypic variability of the syndrome. Our patient also exhibited hearing loss, a common feature reported in H syndrome. However, the conductive nature of hearing loss in this case contrasts with the sensineural hearing loss typically described in the literature, suggesting a potentially unique disease expression.

Polyneuropathy and hearing loss are the most common neurological symptoms reported in the literature of H syndrome,^4^ Both of which were observed in our patient. Additionally, the meningeal enhancement noted on MRI is an infrequent finding, and cerebral edema has been reported in only 5% of cases.^2^ While this finding has not been definitively linked to H syndrome, it may reflect underlying inflammatory or fibrotic processes affecting the meninges.

Several conditions may mimic H syndrome. POEMS syndrome was considered, but the absence of monoclonal gammopathy and organomegaly ruled it out. Rosai–Dorfman disease was excluded due to absence of massive lymphadenopathy and S100+ histiocytes. Langerhans cell histiocytosis was also considered, but lack of CD1a positivity and Birbeck granules, together with the systemic features in our patient, favored H syndrome.

The primary approach for treatment involves addressing the inflammatory component with corticosteroids and immunosuppressive agents, while providing supportive care for systemic complications.^3^

The patient was managed symptomatically with insulin therapy for diabetes, prednisolone for autoimmune hepatitis, and hormone replacement for hypopituitarism. For systemic inflammatory manifestations, corticosteroid therapy was continued. Supportive measures included management of neuropathy and monitoring of lymphadenopathy. No standardized treatment protocol for H syndrome exists, and management remains individualized.

Genetic confirmation of the SLC29A3 mutation and immunohistochemical staining could not be performed due to limited resources. Nevertheless, the clinical, radiological, and histopathological findings strongly supported the diagnosis.

## Conclusion

H syndrome is an extremely rare entity with only 130 reported cases to date. We present this case to raise awareness of this unique syndrome.

## Methods

The case has been reported in accordance with the Surgical Case Report (SCARE) 2023 criteria.^5^

## Conflicts of interest

None disclosed.
